# Clinical characteristics and morbidity among hospitalized adults with advanced HIV disease in Uganda during ‘test and treat’ era

**DOI:** 10.1371/journal.pgph.0002457

**Published:** 2023-10-18

**Authors:** Lillian Tugume, Fred C. Semitala, Darius Owachi, Enock Kagimu, Moses R. Kamya, David B. Meya

**Affiliations:** 1 Department of Medicine, Makerere University College of Health Sciences, Kampala, Uganda; 2 Kiruddu National Referral Hospital, Kampala, Uganda; 3 Makerere University Joint AIDS Program, Kampala, Uganda; 4 Department of Research, Infectious Diseases Institute Makerere University, Kampala, Uganda; PLOS: Public Library of Science, UNITED STATES

## Abstract

Nearly four decades after the first case of AIDS was described, the global number of AIDS-related deaths has steadily declined but falls short of the elimination targets, especially in sub-Saharan Africa. Despite interventions to promote early HIV diagnosis and treatment, hospitalization and mortality related to advanced HIV disease (AHD) remains a significant public health problem in Uganda. We assessed the HIV treatment history and causes of hospitalization among in-patients with AHD at a tertiary hospital in Uganda. In this cross-sectional study, pre-hospitalization HIV treatment history and clinical characteristics of HIV-positive in-patients with CD4<200 cells/μL or WHO stage 3 or 4 clinical events were assessed. Descriptive data were summarized using percentages and medians. Among hospitalized adults with AHD from November 2021 to June 2022, 74% (260/353) knew their HIV status prior to hospitalization and 62% (219/353) were ART experienced at presentation. The median time since ART initiation was 28 months (IQR; 2–97). Overall, 73% (258/353) had at least two etiological diagnoses and the majority (non-mutually exclusive) were diagnosed with tuberculosis (61.2%), cryptococcal meningitis (20.7%), mucosal candidiasis (16.1%) and bacterial infections (15%). In conclusion, nearly two-thirds of in-patients with advanced HIV disease were ART experienced prior to hospitalization and tuberculosis was the most common cause of hospitalization. Innovative strategies to strengthen HIV diagnosis, linkage, and retention in HIV care and to increase coverage of TB preventive therapy are urgently needed.

## Introduction

Access to early HIV treatment has improved markedly since 2015 following the global commitment to end the AIDS epidemic by 2030, through the UNAIDS 90-90-90 and subsequent 95-95-95 targets [1]. In 2016, the WHO recommended providing ART to all people living with HIV irrespective of WHO clinical stage and CD4 count [2]. This ‘test and treat’ strategy additionally includes ART initiation on the same day of HIV diagnosis for those ready to start and in whom immediate ART initiation is not contra-indicated [3, 4]. As a result, countries have made significant gains towards ending AIDS deaths, but the slow rate of mortality decline is concerning [5].

While it is clear that the number of AIDS-related deaths, a measure of Advanced HIV disease (AHD) burden in sub-Saharan Africa is not declining as rapidly as expected the reasons for this in an era of universal HIV treatment and expanded access to ART are less clear. One plausible explanation is missed opportunities for early testing and/ or linkage to care [6, 7]. Alternatively, people living with HIV (PLHIV) who are successfully tested, linked to care, and initiated on ART may be lost to follow-up in a fragile healthcare system and subsequently present to in-patient care facilities with opportunistic infections typical of AHD. In fact, up to a third of HIV-positive adults who are successfully initiated on ART in urban public health facilities in Uganda are lost to follow-up [8–10]. Others may subsequently develop treatment failure often aggravated by a delayed switch to an effective ART regimen thus progressing to AHD [11–13]. Describing in-patients with AHD in terms of their prior continuum of HIV care and the disease syndromes they present with is a critical step towards understanding why advanced HIV disease remains a challenge in the era of universal HIV treatment.

Hospitalized patients with AHD face a greater mortality risk due to opportunistic infections such as tuberculosis [14–16] than outpatients, yet these patients are often underrepresented in studies that have shaped WHO AHD management guidelines [4, 17–19]. In this observational study, we aimed to describe the HIV treatment history and causes of hospitalization among hospitalized patients with Advanced HIV disease at a tertiary hospital in Uganda in the test and treat era.

## Methods

### Study design and setting

Between November 2021 and June 2022, we conducted a cross-sectional study in which we enrolled hospitalized HIV-positive adults from Kiruddu National Referral hospital (Kiruddu hospital) in Uganda. Kiruddu hospital is a 200-bed capacity hospital located ten kilometers from the center of Kampala, the capital City of Uganda. The hospital has a high in-patient turnover with approximately 200 HIV- related admissions per quarter. Of these, up to 80% present with advanced HIV disease [20].

### Study population and participant recruitment

We screened hospitalized HIV-positive patients admitted on the medical wards using CD4 or clinical criteria for advanced HIV disease [21]. Patients were eligible if they were; 18 years or older, with CD4+ <200 cells/μL or WHO clinical stage 3 or 4 disease [22]. Absolute CD4 count was measured using the PIMA CD4 analyzer (Alere, Germany) and HIV testing algorithm followed the Uganda guidelines [23]. Eligible patients were enrolled within 72 hours of hospitalization.

At enrolment, participants or next of kin were interviewed to obtain information on socio-demographics and HIV treatment history prior to hospitalization, including adherence to ART. Adherence was assessed using self-reported number of days missed during the one-month period prior to hospitalization. Missing at least two days in a month was equivalent to 93% adherence yet adherence of 95% or more is associated with optimal virologic outcomes [24]. We assumed that people generally under report poor adherence therefore participants or caretakers who reported missing at least two days in the month prior to admission were considered as sub-optimal adherence. Next of kin were interviewed in cases where the study participants had altered mental status (Glasgow Coma Score<15). To minimize social desirability bias from self-reported HIV treatment history, we used non-judgmental questions to ask about previous HIV treatment history. Vital signs including temperature, respiratory rate, pulse rate and Glasgow coma score were measured by the study team whereas routine laboratory results were abstracted from patient charts. A follow-up chart review on the day of discharge or death was used to collect data on final in-patient diagnosis and date of discharge or death. Diagnoses were determined by the attending clinical care teams. TB diagnosis was categorized as bacteriologically confirmed in the presence of any positive mycobacterial test including Gene Xpert MTB/RIF Ultra or urine lipoarabinomannan (LAM) and as clinically diagnosed in the absence of at least one positive mycobacterial test.

### Data management and analysis

A pre-tested case report form was used to collect demographic (age and sex) and clinical data (HIV treatment history including antiretroviral (ART) status, duration on ART before admission, ART regimen and adherence, baseline vitals, baseline lab parameters including CD4 count, hemoglobin concentration, serum creatinine, random blood sugar and serum albumin). Laboratory values were graded using the Common Terminology Criteria for Adverse Events (CTCAE) Version 5.0 Published: November 27, 2017. Data were entered into Open Data Kit (ODK) then imported into Stata version 13 (StataCorp. 2013. Stata Statistical Software: Release 13. College Station, TX: StataCorp LP) for analysis.

Clinical data were compared by ART status at admission (ART experienced versus ART naïve) using chi-square or Fisher’s exact and Wilcoxon-Mann Whitney test for categorical variables and continuous variables respectively. Further, we compared ART regimen at admission, adherence level in the month preceding admission and proportion of men across three ART duration categories. ART duration categories were defined by the duration of antiretroviral therapy at admission (less than three months, three to six months, and greater than six months). The six months cut-off was selected because virological suppression is expected by six months of consistent ART use and sooner (by three months) with dolutegravir-based regimens. At the time of study implementation, dolutegravir had been widely rolled out and most ART-experienced participants were expected to be on dolutegravir.

Regarding in-patient diagnoses other than TB, conditions with a prevalence of less than 1% of the study population were aggregated as ‘other.’ To account for duplicates and minimize the number of categories, some diagnoses were aggregated as follows:1) alcohol intoxication and alcohol withdrawal as alcohol-related central nervous system (CNS) syndrome, 2) oral candidiasis and esophageal candidiasis as mucosal candidiasis, 3) bronchopneumonia (without a TB diagnosis), sepsis, urinary tract infection, community-acquired pneumonia, empyema thoracis and pelvic inflammatory disease as bacterial infections, 4) acute kidney injury and chronic kidney disease as renal dysfunction and, 5) HIV encephalopathy, HIV induced psychosis, clinically diagnosed viral meningoencephalitis as unspecified encephalopathy.

### Ethical considerations

All study participants or legal representatives provided written informed consent. Ethical approval was obtained from the Makerere University School of Medicine Research and Ethics Committee (Mak-SOMREC-2021-110).

## Results

From November 2021 to June 2022, 353 participants with advanced HIV disease were enrolled and over half, 55% (195/352) were male and the overall median age was 37 years (IQR; 31–45). The median age of men was 40 years (IQR; 33–48) whereas that of women was 34 years (IQR; 29–41). **Table 1** shows the overall summary of clinical characteristics of the study population and comparison by ART status at admission. Of note, ART experienced individuals had significantly higher median CD4 count than those who were ART naïve; 101 cells/μL (IQR29-187) versus 32 cells/μL (IQR:9–102), P<0.001.

**Table 1:** Characteristics of hospitalized adults with advanced HIV disease disaggregated by antiretroviral therapy status prior to hospitalization.

Variable	No. with data	Overall [n(%) or median(IQR)]	ART naïve [n(%) or median(IQR)]	ART Experienced [n(%) or median(IQR)]	P-value^a^
**Age, years**	347	37 (31–45)	35 (30–42)	39 (32–47)	0.049
**Male**	352	195 (55)	78 (59)	117 (53)	0.339
**Weight, Kgs**	325	53 (47–60)	52 (45–60)	54 (47–60)	0.662
Temperature, °C	338	36.8 (36.3–37.5)	36.8 (36.4–37.5)	36.8 (36.3–37.4)	0.476
**GCS <15**	348	93 (27)	24 (23)	47 (30)	0.570
**q-SOFA score> = 2**	348	72 (21)	22 (22)	31 (20)	0.645
**CD4 count, cells/μL**	340	66 (17–167)	32 (9–102)	101 (29–187)	< .001
**Hb, g/dL**	332	10.3 (7.9–12.5)	10.5 (8.2–12.7)	10.1 (7.7–12.3)	0.427
Platelet count, 10^3^/μL	325	198 (127–278)	185 (111–274)	207 (129–291)	0.181
ANC x10^3^/μL	327	3.4 (2.1–5.6)	3.6 (2.0–6.1)	3.3 (2.1–5.2)	0.539
**Serum Creatinine, μmol/L**	301	72.3(55.2–106.2)	70.6 (55.0–94.7)	72.9 (55.6–126.2)	0.406
**Serum Albumin, mg/dL**	274	27.5 (21.5–33.1)	27.3(21.6–32.9)	28.6(21.3–33.2)	0.596

^a^P-value for Chi-square test/ Fisher Exact for categorical variables or Wilcoxon-Mann Whitney test for continuous variables. Abbreviations: ANC-Absolute Neutrophil Count, GCS-Glasgow Coma Scale, Hb-Hemoglobin concentration, Q-SOFA-Quick sequential organ failure assessment.

### HIV treatment history

**Fig 1:**
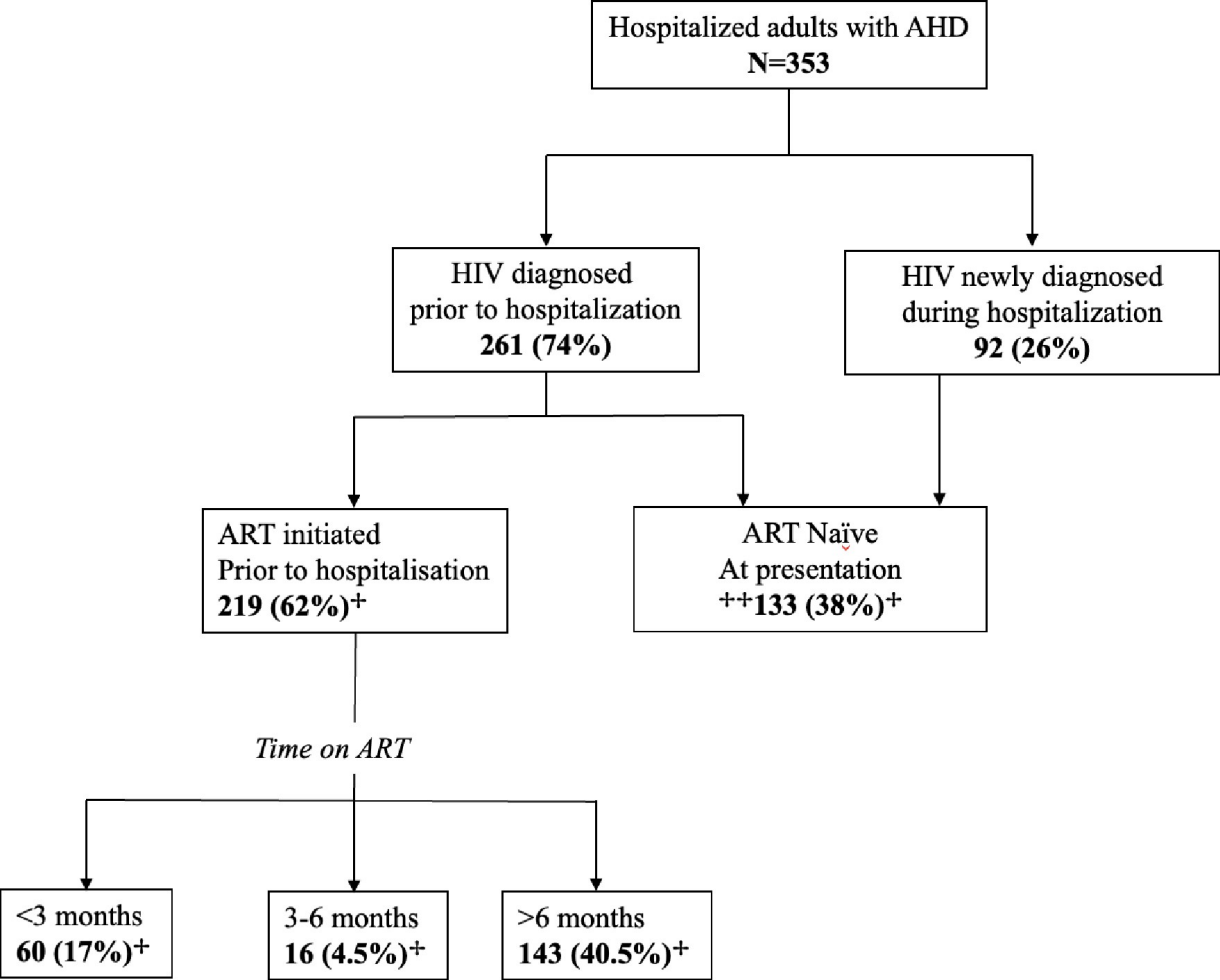
Prior HIV care for hospitalized patients with advanced HIV disease. Abbreviations: AHD- advanced HIV disease, ART- antiretroviral therapy. ^+^ Denominator is all hospitalized adults with advanced HIV disease. ^++^ Includes 92 patients who were newly diagnosed with HIV during hospitalisation and 41 who were aware of HIV status prior to hospitalisation. ART status of one participant cloud not be determined.

Nearly two thirds of study participants; 62% (219/353) were ART experienced and the median time since ART initiation was 28 months (IQR: 2 months to 8 years). Proportions of ART-experienced participants across ART duration categories are illustrated in Fig 1. Among ART-experienced participants whose ART regimen was known, 66% (122/185) were receiving a dolutegravir-based ART regimen and those with more recent ART initiation were more likely to be on a dolutegravir-based regimen. Table 2 illustrates additional aspects of HIV treatment history such as reported adherence and CD4 count across ART duration categories. Among 219 patients who were ART experienced at admission, (30%) reported missing two or more days of ART (suboptimal adherence) in the month preceding admission. Adherence for 10% (23/219) could not be determined. Of note, 17% (60/353) of the study population were on ART for less than 3 months prior to hospitalization and the median time on ART was 0.8 months (3 weeks); with 32% (15/60) of this group reporting suboptimal adherence. The proportions of participants on ART for three to six months and more than six months prior to hospitalization were 5% (16/353) and 40% (143/353) respectively. Thirty six percent (78/219) of ART-experienced participants interrupted ART for one or more months before hospitalization.

**Table 2:** HIV treatment history of ART-experienced individuals with advanced HIV disease.

	Duration on ART prior to hospitalization			
**Variable**	<3 months N = 60	3–6 months N = 16	>6 months N = 143	P-value^b^
**Duration, months**	0.8(0.4–1.5)	4 (3–5)	62(28–134)	
ART regimen^a^				
**Dolutegravir based, n (%)**	56(93)	11(69)	55(38)	< .001
**NNRTI based, n (%)**	0	1(6)	22(15)	< .001
Protease inhibitor-based^c^, n (%)	0	1(6)	16(11)	0.002
**Unknown ART regimen, n (%)**	4(7)	3(19)	50(35)	< .001
Suboptimal adherence^d^, n (%)	15 (32)	3(21)	48(50)	0.038
**CD4 count, median (IQR)**	63 (26–145)	82 (27–213)	114 (35–209)	0.102

^a^ ART regimen before hospitalization.

^b^ P-value for Chi-square or Fisher exact or Kruskal Wallis tests.

^C^ Protease inhibitor regimen is a second line regimen.

^d^Suboptimal adherence is defined based on self-report of missing two or more doses of ART in the 30-day period prior to hospitalization. Percentages are column percentages. Abbreviations: ART- Antiretroviral Therapy; NNRTI- Non-nucleoside Reverse Transcriptase Inhibitor.

Thirty-eight percent (133/353) of the study population were ART naïve at the time of hospitalization (Fig 1). Of these, 32% (42/133) had been diagnosed with HIV prior to admission. Among patients who were aware of their positive HIV status but had never initiated ART, (40/42) 96% were diagnosed at a health facility and the median time from HIV diagnosis to hospitalization was 6 months, IQR (23 days-29 months).

### In-patient diagnosis

**Fig 2:**
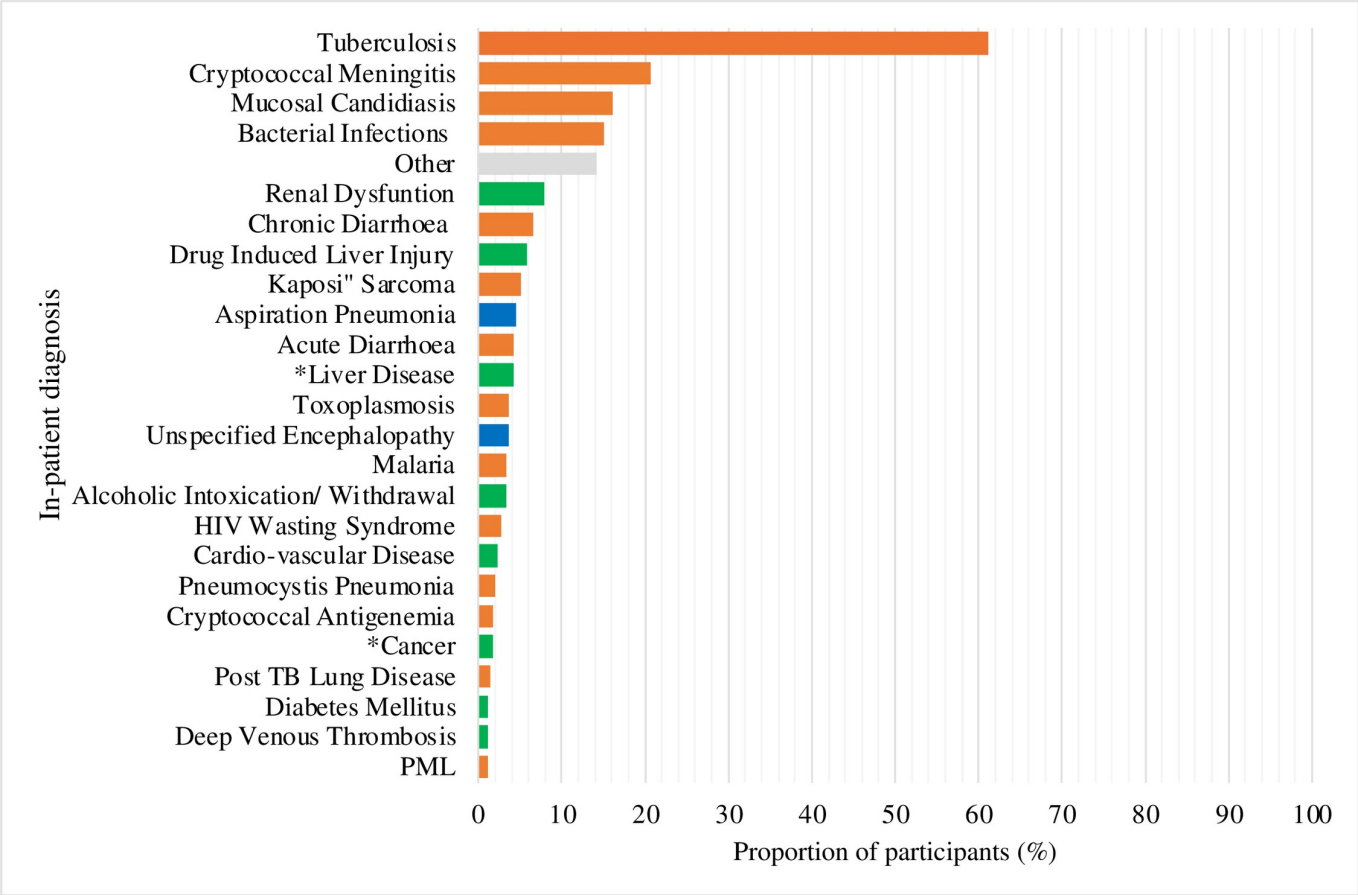
Diagnoses of hospitalized patients with advanced HIV disease. Abbreviations: PML- Progressive Multifocal Leukoencephalopathy. * Liver Disease excludes drug-induced liver injury and liver cancer. **Cancers includes cervical, esophageal, hepatocellular and rhabdomyosarcoma. Data are non-mutually exclusive.

Seventy three percent of participants (257/353) had more than one diagnosis, and 2% (7/353) were unclassified. All unclassified participants died during hospitalization. TB accounted for the majority (61.2%; 216/353) of admissions followed by cryptococcal meningitis (20.7%), mucosal candidiasis (oral, pharyngeal, and esophageal) (16.1%) and bacterial infections (15%). The prevalence of other diagnoses is summarized in **Fig 2**.

Tuberculosis was bacteriologically confirmed in 71% (153/216) of participants and clinically diagnosed in 29%(63/216). Among participants diagnosed with bacteriologically confirmed TB, the basis of diagnosis was positive Urine LAM only in (78%(119/153),), followed by sputum Xpert MTB/RIF Ultra (9%; 13/153), urine LAM combined with urine Xpert MTB/RIF Ultra (8%; 12/153), urine Xpert MTB/RIF Ultra only (5%; 8/153) and lastly, cerebrospinal fluid Xpert MTB/RIF Ultra (0.7%; 1/153). Among patients with clinically diagnosed tuberculosis, the basis of diagnosis was symptoms combined with an abnormal chest X-ray in 57% (36/63). Thirteen percent (27/216) of all TB diagnoses were based on symptoms and signs only and chest X-ray was either not done or was reported as normal.

## Discussion

### Summary of main findings

In this study, we characterized HIV testing and treatment history and co-morbidities of hospitalized patients with AHD in Uganda.

First, we observed that nearly two thirds of in-patients with advanced HIV Disease were ART experienced and presented with a relatively higher CD4 count compared to ART naïve patients. Presentation to in-patient care with advanced HIV disease and prior ART exposure could be attributed to retention failure, immune reconstitution inflammatory syndrome (IRIS) or initiation of ART with profound immune suppression. This is similar to what was observed recently among hospitalized patients with AHD elsewhere in sub-Saharan Africa where the majority were ART-experienced [25].

Regarding individuals who were ART naïve at presentation, 68% were unaware of their HIV status indicating system failures to test some populations early. Contrarily, 32% had been previously diagnosed with HIV but never initiated ART, suggesting failure of linkage to ART initiation.

Finally, we observed that tuberculosis is the commonest co-morbidity in the in-patient setting suggesting that innovative implementation strategies for TB preventative therapy in this population are needed.

### ART-experienced individuals hospitalized with advanced HIV disease

ART-experienced individuals presenting with AHD may have treatment failure, or immune reconstitution inflammatory syndrome or sub optimal immune reconstitution after ART initiation [26, 27]. We hypothesize that patients who were receiving ART for less than 3 months prior to hospitalization (17% of the study population) may not have received sufficient antiretroviral therapy to allow for immune reconstitution because half of the patients in the ‘under three months’ category were receiving ART for only three weeks and one-third reported suboptimal adherence. Thus, patients in the ‘under three months’ category may have had incomplete immune reconstitution after ART initiation and this phenomenon has been described previously among individuals initiating ART at very low CD4 count [27, 28]. Thus, early HIV diagnosis followed by timely ART initiation among populations likely to be missed by existing strategies should be strengthened.

A relatively small proportion (Five percent) of the study population were receiving ART for 3 to 6 months (median time of 4 months) prior to hospitalization and less than a quarter (21%) reported suboptimal adherence. We hypothesize that this ‘3 to 6 months’ category received sufficient ART for immune reconstitution and may be categorized as possible immune reconstitution inflammatory syndrome (IRIS) [26]. The proportion of possible IRIS (5%) is similar to that found in a large prospective cohort of 498 patients in South Africa with rigorous ascertainment and assessment of IRIS events [29] suggesting that contribution of IRIS to hospitalization is likely minimal.

The third category of ART experienced patients were receiving ART for six months or more (40% of our study population) and half reported sub-optimal adherence, therefore we suggest that this population may be classified as possible treatment failure and/or ART non-adherence. Beyond six months of effective ART, viral suppression and immune recovery are expected except in a few isolated cases of sub optimal CD4 recovery despite virological suppression [28, 30]. Of note is that dolutegravir-based regimen was reported for only 66% of individuals whose ART regimen was known, suggesting system failure to achieve near universal switch to dolutegravir- based regimen. Therefore, among ART-experienced patients hospitalized with advanced HIV disease, we believe that treatment failure and poor adherence accounted for most admissions. Long-acting ART formulations should be considered in this sub-population.

### ART naïve individuals hospitalized with advanced HIV disease

Over a third of the study population were ART naïve at presentation, indicating gaps in early HIV diagnosis and linkage to care. The majority of ART naïve patients were not aware of their HIV status prior to hospitalization, and this implies that lack of early HIV diagnosis remains a barrier of eliminating AHD. Similarly, the 2022 UNAIDS report indicated that no country in sub-Saharan Africa was able to meet the ‘first 95% target’ for 95% of all people living with HIV knowing their HIV status [31]. This calls for strengthening strategies for testing individuals that are difficult to reach such as assisted partner notification, and targeted testing among high risk and high incident populations. Among populations where these high yield testing approaches have already been widely implemented, a strategic mix of differentiated HIV testing services irrespective of yield may be required to further improve case finding [32, 33].

### Causes of hospitalization

Our study suggests that tuberculosis accounts for most of the morbidity associated with advanced HIV disease among in-patients. This is consistent with what has been observed elsewhere in sub-Saharan Africa, however the prevalence of TB in our study was relatively higher than expected (61% versus 40%) [34, 35]. This could be due to true increased prevalence related to health systems disruption by the COVID-19 pandemic or improved diagnostic capacity [36]. Notably, four of every five tuberculosis diagnoses were bacteriologically confirmed, and this is probably attributed to the expanded access to urine LAM, which is highly specific for diagnosis of TB among hospitalized patients with AHD [37]. Therefore, we suggest that TB preventative therapy (TPT) should be strengthened among individuals with advanced HIV disease.

TB preventative therapy is recommended by WHO for all people living with HIV (PLHIV) with a target of 90% of all PLHIV by 2025, however implementation is suboptimal globally [38, 39]. Among individuals with advanced HIV disease, implementation may be limited partly by inability to exclude active TB in ill patients and urinary TB diagnostics such as LAM may foster initiation of TPT and early TB treatment. In particular, TB symptoms may overlap with symptoms of systemic mycoses such as cryptococcosis which is highly prevalent in AHD population in Uganda [40]. It is not uncommon for TB and cryptococcosis to occur in the same patient [40]. TPT is further limited by high pill burden and poor medication adherence, thus, shorter regimens such as one month of Isoniazid and Rifapentine are preferred in the advanced HIV disease population [41, 42]. System barriers such as drug stock outs, clinician knowledge gaps, and insufficient TB‐dedicated staff also contribute to the low TPT uptake in Uganda [41].

### Limitations

The main limitation of this observational study is related to the assessment of HIV treatment history. ART status and adherence were assessed by self-report, potentially leading to social desirability bias and risk of misclassifying individuals who are ART experienced as ART naïve. This bias was mitigated by asking the question in a non-judgmental manner with preceding empathetic statements. Additionally, the assessors were not directly involved in clinical care of the study participants. Our findings showed that ART-experienced patients had higher CD4 counts than ART naïve, therefore we believe that the assessment of ART status was reliable.

Second, in-patient diagnoses were collected by chart review and data were insufficient to categorize diagnoses as confirmed versus clinical/ presumptive. This was only possible for TB diagnosis, therefore our discussion on causes of hospitalization is focused on TB which is also the most prevalent.

Finally, documentation of viral loads was challenging because attaining a viral load required a linked clinic ID, which many participants did not have. Viral load testing would have complemented interpretation of HIV treatment history, which is important for diagnosis of treatment failure and IRIS. For instance, diagnosis of IRIS was challenging in the absence of viral load as IRIS can occur across all three ART duration categories. Patients with unmasking IRIS more likely in the <3 months group, those with unmasking/paradoxical IRIS could fall in the 3–6-month group and some patients in the >6months group could have paradoxical IRIS. Considering challenges in access to plasma viral load, we recommend that point-of-care viral load testing should be added to the advanced HIV disease package of care for hospitalized patients.

## Conclusion

In summary, our findings suggest that the prevalence of advanced HIV disease among hospitalized adults is attributable to clinical failure reflected by opportunistic infections and poor adherence in a predominantly ART-experienced population. Slow change to near universal DTG regimen may have contributed to treatment failure. Long-acting ART formulations such as 2-monthly cabotegravir plus rilpivirine should be evaluated in effectiveness trials for ART experienced adults with suboptimal virological control. This regimen may improve adherence and modelling studies have shown low risk of emergent resistance [43]. Additionally, delayed HIV diagnosis and initiation of antiretroviral therapy still occur in the “test and treat” era, therefore, programs should additionally target populations that are likely to be missed by existing testing strategies. We hypothesize that immune reconstitution inflammatory syndrome was less likely to be a major cause of hospitalization, however, this remains subject to further research. Finally, most admissions were attributed to tuberculosis, and we recommend that TPT should be strengthened in advanced HIV disease programs.

## Supporting information

S1 Dataset Microsoft Excel file of the data set. (XLS)

S1 File Stata “do” file used for data analysis. (DO)

S2 File Microsoft Excel file used to generate Fig 2. (XLSX)
